# PPAR-γ Inhibits Chronic Apical Periodontitis by Facilitating Macrophage Efferocytosis

**DOI:** 10.3390/ijms262010157

**Published:** 2025-10-19

**Authors:** Yuting Wang, Mingfei Wang, Xiaowen Jia, Yifei Tang, Jiayi Wang, Wenjiao Zhang, Tiezhou Hou, Xiaoyue Guan

**Affiliations:** 1 Key Laboratory of Shaanxi Province for Craniofacial Precision Medicine Research, College of Stomatology, Xi’an Jiaotong University, Xi’an 710049, China; 2 Clinical Research Center of Shaanxi Province for Dental and Maxillofacial Diseases, College of Stomatology, Xi’an Jiaotong University, Xi’an 710049, China; 3 Department of Cariology and Endodontics, College of Stomatology, Xi’an Jiaotong University, Xi’an 710049, China

**Keywords:** apical periodontitis, PPAR-γ, macrophage, efferocytosis

## Abstract

This study aimed to elucidate the role of peroxisome proliferator-activated receptor-γ (PPAR-γ) in regulating macrophage efferocytosis during the pathogenesis of chronic apical periodontitis (CAP). Clinical specimens, rat periapical lesion models, and an in vitro model simulating the CAP inflammatory milieu were employed to examine the contribution of PPAR-γ to efferocytosis throughout disease progression. The expression of PPAR-γ in vivo was assessed by single-cell RNA sequencing and immunohistochemical (IHC) staining. Pearson’s correlation and linear trend tests were conducted to investigate the association between PPAR-γ and macrophage efferocytosis during CAP progression. Pharmacological modulation of PPAR-γ was further conducted using rosiglitazone (RSG) as an agonist and GW9662 as an antagonist, followed by an assessment of efferocytosis-related parameters and inflammatory responses. Both clinical specimens and animal models demonstrated a progressive reduction in PPAR-γ expression and macrophage efferocytosis during CAP. Notably, PPAR-γ attenuated efferocytosis impairment and significantly reduced pathogen-induced inflammatory responses in macrophages. These findings indicate that defective macrophage efferocytosis contributes to the exacerbation of CAP severity, whereas targeting PPAR-γ may represent a promising therapeutic strategy to alleviate inflammation in periapical lesions by restoring efferocytic capacity. Collectively, this study highlights PPAR-γ as a potential therapeutic target warranting further investigation in CAP treatment.

## 1. Introduction

**Figure 1:**
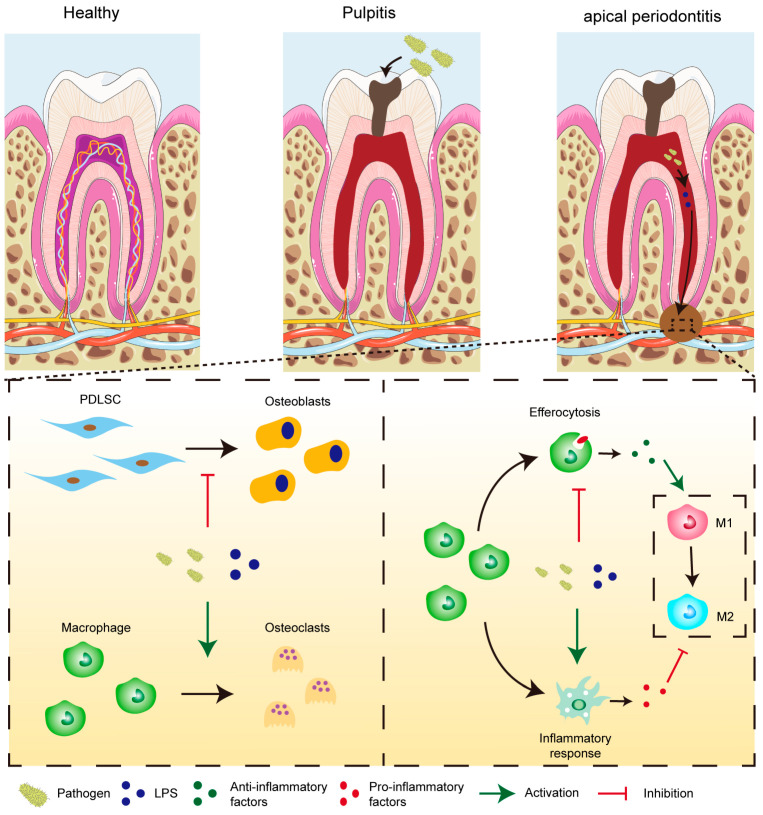
Pathophysiology of apical periodontitis.

Chronic apical periodontitis (CAP) is a prevalent and progressive oral pathology characterized by the resorption of periapical alveolar bone (Figure 1) [1,2]. Epidemiological studies showed that about 52% of the global adult population has at least one tooth with periapical periodontitis [3]. This chronic inflammatory condition arises from a complex interaction among microbial agents, inflammatory mediators, and immune responses, ultimately resulting in alveolar bone loss and tooth retention challenges, with potential systemic implications [4,5]. Conventionally, root canal treatment (RCT), which involves thorough debridement followed by hermetic sealing of infected root canal spaces, is the primary treatment modality aimed at eradicating intracanal infection and avoiding reinfection. However, it is important to recognize that approximately 4% to 15% of treated teeth may experience persistent pain necessitating extraction post-RCT [6,7]. Furthermore, accumulating evidence has linked CAP with systemic conditions such as cardiovascular disease, rheumatoid arthritis, depression, and anxiety, underscoring the urgent need to develop novel and effective therapeutic strategies for CAP [8].

**Figure 2:**
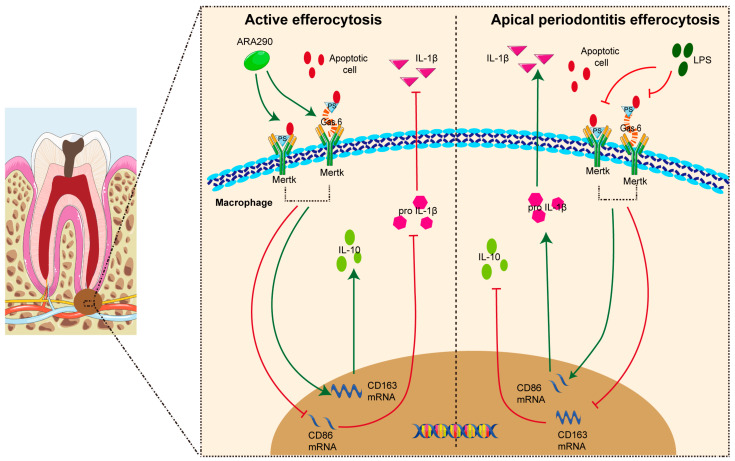
Mechanisms and molecular implications of macrophage efferocytosis in CAP.

Macrophages, as essential components of the innate immune system and the primary defense against microbial invasion, play a vital role in the progression of CAP [9]. Growing evidence underscores the importance of macrophage efferocytosis—the process by which macrophages, known as efferocytes, remove apoptotic neutrophils from inflamed tissues, thereby influencing their polarization state [10,11]. Upon engulfment of apoptotic cells, efferocytes secrete a spectrum of bioactive mediators and extracellular vesicles that can be absorbed by surrounding macrophages, promoting a phenotypic transition toward the anti-inflammatory M2 subtype [12]. Our earlier work revealed that macrophage efferocytosis is progressively impaired as CAP advances, as evidenced in clinical samples [13]. This reduction in efferocytic activity was linked to increased M1 polarization and a higher M1/M2 ratio. Conversely, enhancing efferocytosis facilitated M1-to-M2 conversion and alleviated disease progression [13]. Collectively, these findings indicate that targeting macrophage efferocytosis may represent a promising therapeutic avenue for CAP management (Figure 2).

**Figure 3:**
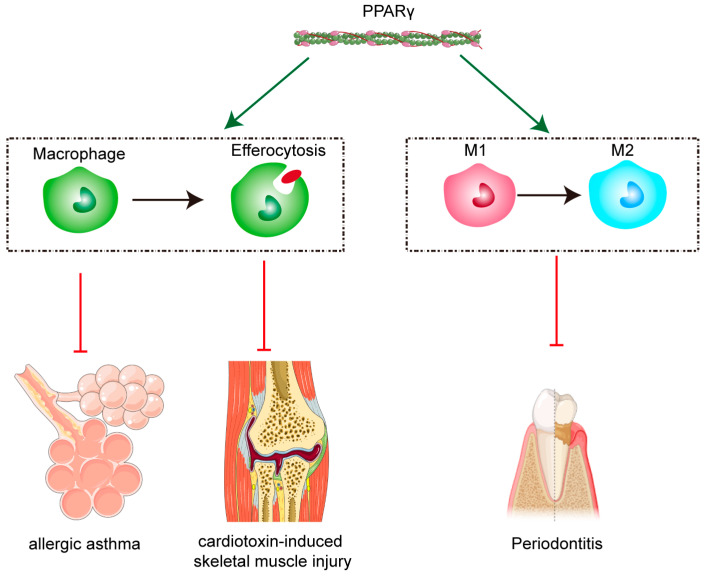
Role of PPAR-γ in various diseases.

Peroxisome proliferator-activated receptor γ (PPAR-γ) serves as a pivotal nuclear transcription factor that governs multiple physiological and pathological pathways, encompassing inflammation, immune modulation, and metabolic regulation [14]. Numerous studies have implicated PPAR-γ in the onset and progression of various inflammation-associated disorders [15,16]. Within oral inflammatory microenvironments, particularly periodontitis, the regulatory influence of PPAR-γ has been extensively explored [17,18]. For example, Wang et al. demonstrated that elevated PPAR-γ expression in inflamed periodontal tissues suppresses tissue destruction, whereas its inhibition intensifies disease progression [19]. Moreover, PPAR-γ has emerged as a crucial regulator of macrophage efferocytosis. Huang et al. revealed that PPAR-γ activation enhances macrophage-mediated clearance of apoptotic cells, thereby alleviating airway inflammation in allergic asthma [20]. Consistently, Garabuczi et al. showed that PPAR-γ upregulation strengthens efferocytosis, drives macrophage polarization toward the M2 phenotype, and accelerates recovery from cardiotoxin-induced muscle injury [21]. Nevertheless, the specific function of PPAR-γ in CAP and its potential modulatory effects on macrophage efferocytosis during CAP development remain largely unexplored (Figure 3).

In the current research, single-cell RNA sequencing was utilized to profile PPAR-γ expression within periapical macrophages isolated from patients diagnosed with CAP. A corresponding CAP animal model was subsequently constructed to explore the association between PPAR-γ levels and the efferocytosis-related receptor Mertk. Furthermore, macrophages with either silenced or overexpressed PPAR-γ were generated to assess their capacity for apoptotic neutrophil clearance, followed by an evaluation of their polarization dynamics. Through these investigations, we sought to clarify the functional involvement of PPAR-γ in CAP progression and to uncover the molecular basis by which it regulates macrophage efferocytosis. Collectively, our data point to PPAR-γ as a potential therapeutic target for the management of CAP.

## 2. Results

### 2.1. Single-Cell Sequencing

**Figure 4:**
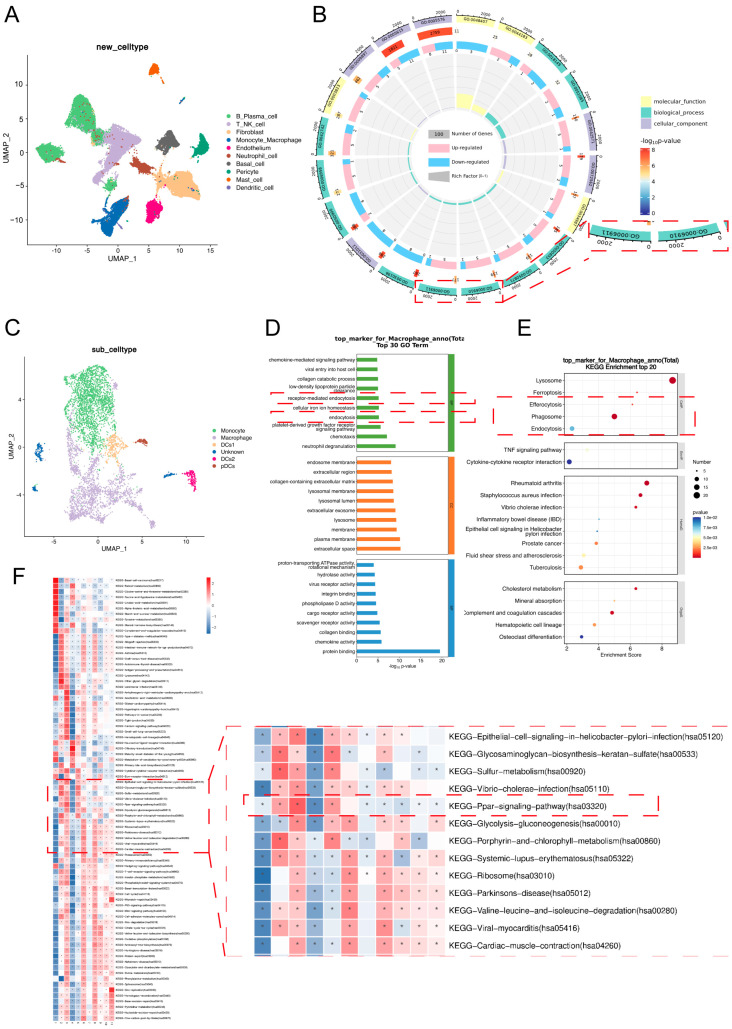
Single-cell sequencing of AP tissue. (**A**) 2D visualization (UMAP dimensionality reduction) of pooled data from scRNA-seq data of AP. (**B**) The GO function enrichment of DEGs between AP and NC samples. The highlighted part was GO0006911 (phagocytosis, engulfment) and GO0006910 (phagocytosis, recognition). (**C**) Identification of macrophage subtypes. (**D**,**E**) KEGG pathway enrichment (**E**) and GO functional enrichment (**D**) of all genes in macrophages. The highlighted part was receptor-mediated endocytosis, endocytosis, efferocytosis, phagosome, and endocytosis. (**F**) GSVA analysis of DEGs in different subtypes of monocyte and macrophage clusters. NC, healthy gingiva tissue, AP, and apical periodontitis, * There is an inter-group difference between the AP group and the NC group, *p* < 0.05.

To investigate the potential mechanisms by which macrophages regulate the progression of apical periodontitis, we analyzed single-cell RNA sequencing (scRNA-seq) data derived from clinical samples, including healthy gingival tissues (NC, *n* = 3) and apical periodontitis tissues (AP, *n* = 3). Unsupervised UMAP clustering, based on transcriptomic profiles, identified multiple immune cell populations in both NC and AP, including monocytes/macrophages, B plasma cells, T cells, NK cells, and neutrophils (Figure 4A). Functional enrichment analysis of differentially expressed genes (DEGs) revealed that phagocytosis-related biological processes, such as GO:0006911 (phagocytosis, engulfment) and GO:0006910 (phagocytosis, recognition), were among the key pathways potentially regulating apical periodontitis progression (Figure 4B). Notably, macrophages were identified as the predominant cell type engaged in phagocytic activity. Subtype analysis of the monocyte/macrophage cluster further confirmed that macrophages constituted the major cellular subset (Figure 4C). To elucidate their functional contribution to disease progression, macrophage functional profiling was performed, which demonstrated significant enrichment in receptor-mediated endocytosis, general endocytosis, efferocytosis, and phagosome-related pathways (Figure 4D). Finally, gene set variation analysis (GSVA) indicated that macrophage functions in apical periodontitis may be mediated, at least in part, through activation of the PPAR signaling pathway (Figure 4E).

### 2.2. The Expression of PPAR-γ Varies Depending on Chronic Apical Periodontitis Development in Clinical Samples

**Figure 5:**
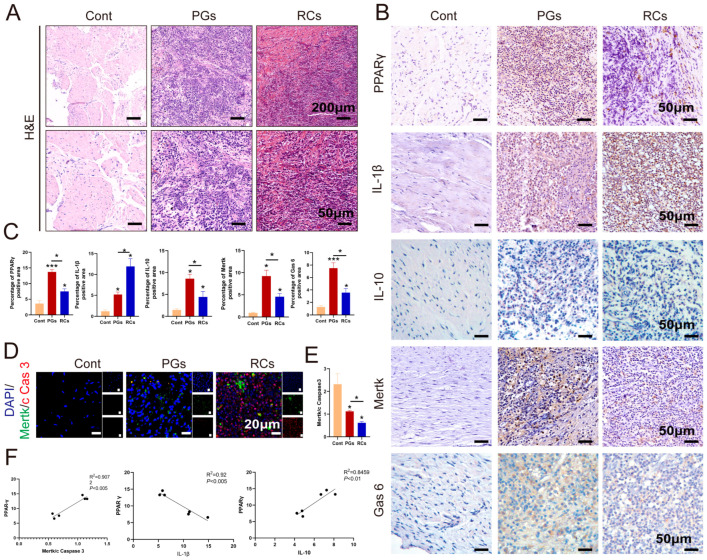
Macrophage efferocytosis and the expression of PPAR-γ in chronic apical periodontitis. (**A**) HE staining of different groups. The upper scale = 200 µm, the lower scale = 50 µm. (**B**,**C**) Expression of PPAR-γ, IL-1β, IL-10, Mertk, and Gas 6 (**B**) and quantification of protein level (**C**) in each group. The scale bar = 50 µm. (**D**,**E**) Co-location between Mertk and cleaved Caspase 3 (**D**) and the ratio between Mertk and cleaved Caspase 3 (**E**) in each group. The scale bar = 20 µm. (**F**) Correlation of PPAR-γ and Mertk/cleaved Caspase 3, IL-1β, and IL-10 in PGs and RCs. Cont, control group, PGs, periapical granuloma, RCs, radicular cyst. c Cas 3 and c Caspase 3, cleaved Caspase 3. * *p* < 0.05, *** *p* < 0.005.

Based on single-cell sequencing of clinical specimens, the potential involvement of PPAR-γ in macrophage efferocytosis was indicated. To further elucidate the regulatory role of PPAR-γ in macrophage efferocytosis within periapical tissues, we examined both macrophage efferocytosis activity and PPAR-γ expression in periapical lesions. Initially, hematoxylin–eosin (HE) staining confirmed the inflammatory status of the clinical samples, demonstrating that periapical tissues with CAP displayed a significantly higher infiltration of inflammatory cells compared with healthy controls (Figure 5A). Subsequently, immunohistochemical (IHC) staining unraveled elevated IL-1β expression in radical cysts (RCs) relative to periapical granulomas (PGs), whereas the expression levels of Mertk, Gas6, and IL-10 were more pronounced in PGs than in RCs (Figure 5B,C). Furthermore, immunofluorescence (IF) co-staining was conducted to assess the co-expression of Mertk and c-caspase-3 within the periapical region. These data indicated a decreased ratio of Mertk to c Caspase 3, particularly in the RCs groups (Figure 5D,E). Following the reconfirmation of macrophage efferocytosis in periapical lesions, PPAR-γ expression was evaluated. Compared with Mertk and Gas6, PPAR-γ expression was notably higher in PGs than in RCs (Figure 5B,C). Correlation analyses between PPAR-γ and IL-1β, PPAR-γ and IL-10, as well as PPAR-γ and Mertk/c-Caspase-3 ratio, revealed a significant negative correlation between PPAR-γ and IL-1β (R2 = 0.92, *p* < 0.005), alongside strong positive correlations between PPAR-γ and IL-10 (R2 = 0.8459, *p* < 0.01), and between PPAR-γ and Mertk/c-Caspase-3 ratio (R2 = 0.9072, *p* < 0.005) (Figure 5F). Collectively, these results suggest that PPAR-γ may be accountable for the reduced phagocytosis of apoptotic neutrophils by Mφs, with its downregulation potentially contributing to the exacerbation of CAP.

### 2.3. The Aggravation of CAP in the Animal Model Was Associated with the Down-Regulation of PPAR-γ and a Sustained Dysfunction in Macrophage Efferocytosis

**Figure 6:**
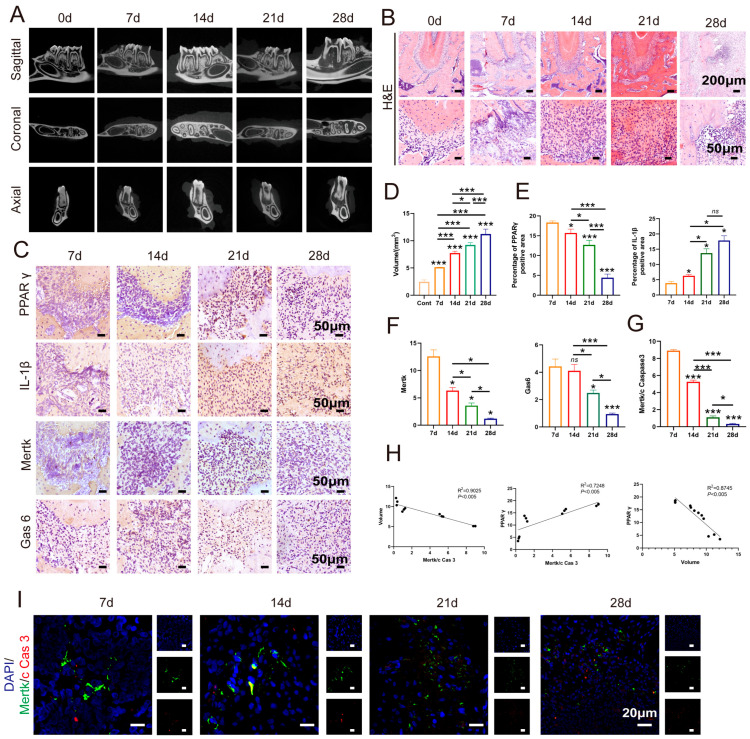
Macrophage efferocytosis and the expression of PPAR-γ in the development of apical periodontitis. (**A**) Representative images of radiological examinations in each group. (**B**) HE staining illustrating the inflammatory infiltrates in the development of apical periodontitis. The upper scale bar = 200 µm, the lower scale bar = 50 µm. (**C**) Immunohistochemical staining of PPAR-γ, IL-1β, Mertk, and Gas 6 in each group. The scale bar = 50 µm. (**D**) Quantification of alveolar bone loss volume in each group. (**E**,**F**) Quantification of PPAR-γ, IL-1β (**E**), Mertk, and Gas 6 (**F**) in each group. (**G**,**I**) Co-location between Mertk and cleaved Caspase 3 (**I**) and the ratio of Mertk to cleaved Caspase 3 (**G**) in each group. (**H**) Correlation of volume and Mertk/c Caspase 3, PPAR-γ and Mertk/c Caspase 3 and PPAR-γ and volume. c Cas 3 and c Caspase 3, cleaved Caspase 3. ns *p* > 0.05, * *p* < 0.05, *** *p* < 0.005.

To further clarify the role of PPAR-γ in modulating macrophage efferocytosis during the progression of CAP, a CAP animal model was established using forty Sprague–Dawley (SD) rats. Following pulp exposure, the animals were euthanized at predetermined intervals on days 0, 7, 14, 21, and 28, with 8 rats assigned to each time point. Micro-CT and HE staining revealed a progressive increase in the volume of bone resorption within the periapical regions over time (Figure 6A,B,D). The mandibles collected on day 0 post-pulp exposure served as the control group. On day 0, the periapical tissues around the root apex remained intact, with minimal inflammatory cell infiltration and no observable bone resorption (Figure 6A,B,D). Alveolar bone loss in the periapical region was first detected 7 days after pulp exposure. Subsequently, bone loss expanded across the horizontal, coronal, and sagittal planes from day 7 through day 28. Additionally, the number of inflammatory cells increased concomitantly with the advancement of CAP in the rats (Figure 6A,B,D).

During the progression of CAP, alveolar bone resorption occurs, serving as an indicator of CAP. Consequently, we conducted further investigations to evaluate the expression levels of the pro-inflammatory cytokine IL-1β and the macrophage efferocytosis markers Mertk and Gas6 and PPAR-γ within the periapical regions affected by CAP (Figure 6C,E,F). Consistent with expectations, IL-1β protein levels exhibited a significant increase from day 7 to day 28 post-pulp exposure (Figure 6C,E). In contrast, the expressions of Mertk, Gas6, and PPAR-γ showed a pronounced decline from day 7 to day 28 (Figure 6C,F). Additionally, co-localization analyses of Mertk and cleaved caspase-3 (c-Caspase-3) were performed. As illustrated in Figure 6I,G, the ratio of Mertk to c-Caspase-3 slightly decreased from 7 days to 28 days after pulp exposure. Furthermore, correlations between PPAR-γ and Mertk/c-Caspase-3 ratio, PPAR-γ and bone loss volume, and bone loss volume and Mertk/c-Caspase-3 ratio were examined. The results revealed a statistically significant positive correlation between PPAR-γ expression and the Mertk/c-Caspase-3 ratio (R2 = 0.7248, *p* < 0.005), as well as robust negative correlations between PPAR-γ and bone loss volume (R2 = 0.8745, *p* < 0.005), and between the bone loss volume and Mertk/c-Caspase-3 ratio (R2 = 0.9025, *p* < 0.005) (Figure 6H). In summary, these findings provide additional evidence underscoring the pivotal role of PPAR-γ in the regulation of CAP progression through the modulation of macrophage efferocytosis.

### 2.4. Interfering with the Expression of PPAR-γ Effectively Modulates Macrophage Efferocytosis in an In Vitro Model Mimicking the Environment of CAP

**Figure 7:**
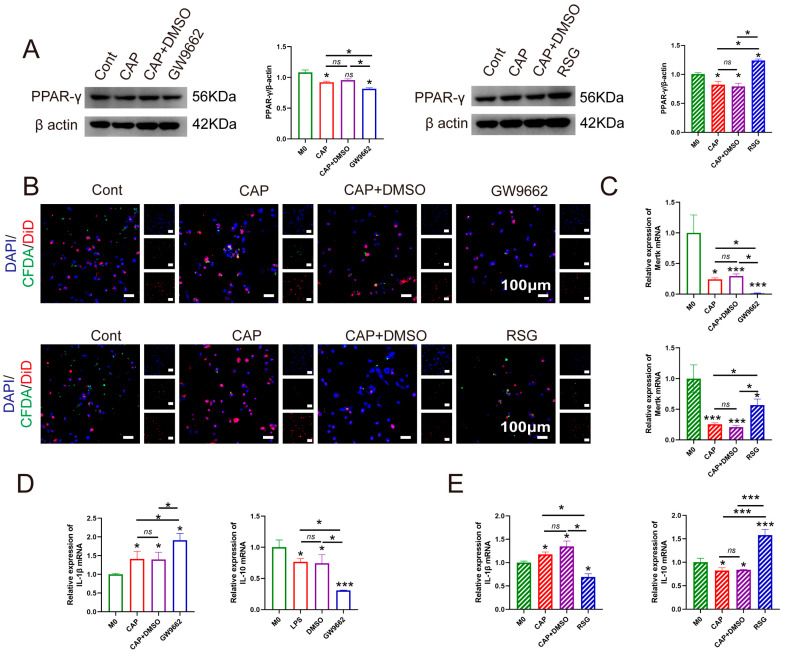
Role of PPAR-γ in regulating macrophage efferocytosis and the progression of apical periodontitis. (**A**) Protein level and the quantification of PPAR-γ in each group. (**B**) Rate of macrophage efferocytosis in each group. Green, apoptotic neutrophils; red, macrophages; blue, DAPI. Scale bar = 100 µm. (**C**–**E**) Expression of Mertk (**C**), IL-1β, and IL-10 (**D**,**E**) in each group was investigated. RSG, rosiglitazone. ns *p* > 0.05, * *p* < 0.05, *** *p* < 0.005.

To clarify the role of PPAR-γ in macrophage efferocytosis during CAP, we simulated an inflammatory milieu using *E. coli* lipopolysaccharide (LPS). Macrophages were treated with the PPAR-γ agonist rosiglitazone (RSG) or the antagonist GW9662. Western blotting confirmed that RSG upregulated, while GW9662 suppressed, PPAR-γ expression (Figure 7A). Immunofluorescence showed that RSG markedly enhanced macrophage efferocytosis of apoptotic neutrophils, whereas GW9662 impaired this process (Figure 7B). Consistently, the efferocytosis-related receptor Mertk was increased by RSG and reduced by GW9662 (Figure 7C). Moreover, RSG treatment lowered IL-1β and elevated IL-10 levels, while GW9662 exerted opposite effects (Figure 7D,E). Together, these findings indicate that PPAR-γ activation promotes macrophage efferocytosis and attenuates inflammation in CAP, whereas its inhibition hampers efferocytosis and aggravates disease progression.

## 3. Discussion

Recent evidence highlights the pivotal role of macrophage efferocytosis in resolving inflammation, particularly in oral lesions. Our previous findings revealed that promoting macrophage efferocytosis facilitates the remission of CAP, indicating that identifying molecular pathways enhancing this process could improve therapeutic efficacy [13]. Here, we investigated the involvement of PPAR-γ in CAP pathogenesis. Single-cell RNA sequencing confirmed the presence of PPAR-γ in macrophages from periapical tissues of both healthy and CAP subjects (Figure 4), and its expression was positively correlated with efferocytic activity. Analyses of clinical samples and experimental models showed that reduced PPAR-γ expression coincided with diminished macrophage efferocytosis (Figure 5 and Figure 6). Using an in vitro inflammatory model, we demonstrated that PPAR-γ activation increased IL-10 and decreased IL-1β production by enhancing efferocytosis (Figure 7), providing the first evidence that PPAR-γ regulates macrophage efferocytosis in periapical lesions and may serve as a therapeutic target for CAP.

During CAP, macrophages undergoing M1 polarization amplify inflammation by secreting large amounts of pro-inflammatory cytokines [22,23], aggravating tissue destruction, disturbing bone homeostasis, and hindering repair [24,25,26]. In contrast, M2 macrophages secrete IL-10 and promote tissue regeneration [27,28]. Efferocytosis has been shown to induce M2 polarization, reduce inflammatory responses, and accelerate bone regeneration [29,30]. Our prior studies confirmed that enhanced efferocytosis drives M1-to-M2 transition in periapical lesions, lowering IL-1β and increasing IL-10, thereby facilitating inflammation resolution [13]. In this study, single-cell sequencing and IHC validated the presence of macrophage efferocytosis in periapical tissues. Both patient samples and animal models exhibited reduced efferocytic function and heightened inflammation as CAP progressed, emphasizing the need to identify targets that restore macrophage efferocytosis and promote lesion repair (Figure 4, Figure 5 and Figure 6).

Emerging research supports PPAR-γ as a key modulator of macrophage efferocytosis [31]. Garabuczi et al. reported that PPAR-γ upregulation promotes M2-like reparative polarization during skeletal muscle regeneration [21]. Furthermore, Liu, X et al. corroborated the critical role of PPAR-γ in macrophage efferocytosis during inflammatory processes [32]. Specifically, their findings revealed that PPAR-γ activation enhances the clearance of apoptotic cells by macrophages, contributing to the resolution of ischemic stroke in animal models and effectively mitigating white matter injury and cognitive impairment [32]. Existing therapeutic strategies targeting PPAR-γ are presented in Table 1. To further validate these observations, macrophages were treated with the PPAR-γ agonist RSG or the antagonist GW9662. Importantly, our previous work demonstrated that macrophage efferocytic capacity is initially enhanced during early inflammatory responses to counteract tissue damage; however, as CAP advances, this efferocytic function becomes markedly impaired, resulting in the accumulation of apoptotic neutrophils in periapical lesions. This accumulation triggers a cascade of inflammatory events that ultimately leads to progressive alveolar bone loss. In the current study, LPS and IFN-γ were applied to the co-cultured cell model for 12 h to simulate CAP in vitro. (Figure 7). These findings indicate that PPAR-γ enhances macrophage efferocytosis, limits inflammation, and mitigates alveolar bone loss, suggesting its potential as a therapeutic target for CAP management.

This study has several limitations. Due to constraints in clinical specimen acquisition, the number of samples analyzed was relatively small. Although post hoc power analysis indicated adequacy for statistical interpretation, the limited cohort may still restrict the general applicability of our conclusions. Moreover, this work only provides a preliminary exploration of how PPAR-γ influences macrophage efferocytosis in periapical periodontitis, while the detailed regulatory mechanisms remain to be clarified. Future investigations will focus on delineating the upstream and downstream pathways of PPAR-γ signaling in modulating macrophage efferocytosis, thereby establishing a mechanistic framework for clinical translation and the design of novel adjunctive therapeutic strategies to improve treatment outcomes in periapical periodontitis.

In conclusion, our findings demonstrate that activation of PPAR-γ signaling alleviates inflammation and tissue injury in periapical lesions during CAP progression by enhancing macrophage efferocytosis. These results highlight PPAR-γ as a promising therapeutic target for CAP management. Moving forward, we intend to evaluate the adjunctive efficacy of established PPAR-γ agonists, such as rosiglitazone, in treating periapical periodontitis. Furthermore, the development of localized delivery systems—such as sustained-release hydrogels—may facilitate the translational application of this therapeutic strategy.

## 4. Materials and Methods

**Figure 8:**
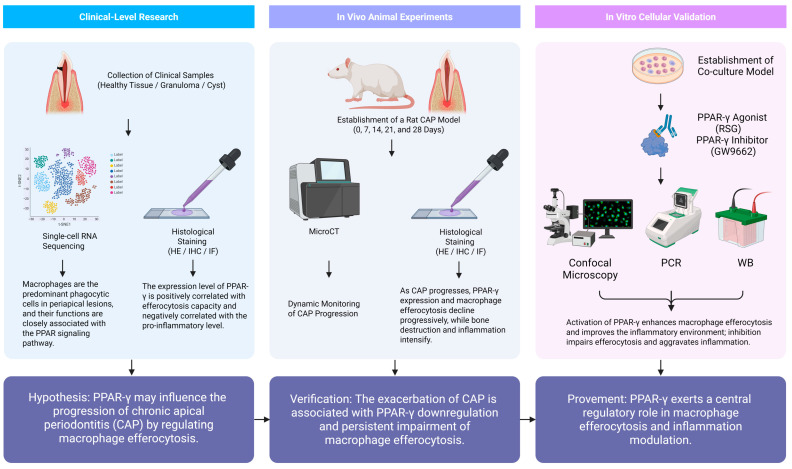
Research methods.

The experimental methods are shown in Figure 8.

### 4.1. Ethical Statements

The experiment protocols of clinical samples and animals received approval from the Medical Ethics Committee of Xi’an Jiaotong University Stomatological Hospital and the Laboratory Animal Ethics Committee of Xi’an Jiaotong University. Prior to the collection of the clinical samples, the participants were clearly informed of the objectives and the content of the research. In addition, all participants signed the consent forms. In addition, the animals were bred in a standard environment (12 h light/dark cycle with stable temperature (25 ± 2 °C) and humidity (50 ± 5%)) and fed with a standard diet and water for one week before the animal experiment.

### 4.2. Clinical Sample Collection and Single-Cell Sequencing

The clinical samples, including healthy gingiva tissues (Cont, *n* = 10), periapical granuloma (PGs, *n* = 10), and radicular cyst (RCs, *n* = 10), were collected from Xi’an Jiaotong University Stomatological Hospital. As shown in Figure S1, the inclusion criteria for AP aligned with previous studies [35]. The sample size was determined based on the previous research [2]. The clinical samples were fixed in 4% paraformaldehyde for 48 h at room temperature. The demographic characteristics of the clinical sample are shown in Table S1.

Then, the clinical samples, including healthy gingiva tissues (NC, *n* = 3) and apical periodontitis tissues (AP, *n* = 3), were collected from Xi’an Jiaotong University Stomatological Hospital. The tissues were used to prepare single-cell suspensions according to the manufactural instructions, and raw data of single-cell RNA was generated as FASTQ files. In addition, the two-dimensional Uniform Manifold Approximation and Projection (UMAP) algorithm, GO and KEGG pathway enrichment analysis of DEGs, and GSVA analysis were performed to analyze the cellular heterogeneity and identify the differentially expressed genes (DEGs).

### 4.3. The Establishment of a CAP Animal Model

A total of 50 male SD rats were purchased from the Laboratory Animal Center, Xi’an Jiaotong University. The protocols of CAP animal model establishment were described in a previous study [2]. First, rats were anesthetized with pentobarbital sodium (3 mg/kg) via intraperitoneal injection. After 20–25 min to guarantee comprehensive anesthesia, the rats in the CAP animal model group were individually positioned on specialized surgical tables for the subsequent procedure. The pulp cavities of bilateral mandibular first molars were exposed via a 1/4# round bur. Following the pulp cavity exposure for 7 d, 14 d, 21 d, and 28 d, the bilateral mandibular first molars were harvested and fixed in 4% paraformaldehyde for 48 h at room temperature. The prognostic and demographic characteristics of the animals are shown in Table S2.

### 4.4. The In Vitro Model Establishment

Human monocytic leukemia THP-1 cells and HL-60 were acquired from Procell Life Science & Technology Co., Ltd. (Wuhan, China), cultured in RPMI-1640 medium (Gibico, Billings, MT, USA) supplemented with 10% fetal bovine serum (FBS) (BI, Kibbutz, Beit Haemek, Israel) and maintained in a humidified environment at 37 °C with 5% CO_2_. Initially, the apoptotic HL-60 cells were induced as previous study. The THP-1 cells were treated with 100 ng/mL PMA (Sigma, Darmstadt, Germany) for 24 h to induce macrophage differentiation. Then, the differentiated THP-1 cells were stimulated with 100 ng/mL LPS and 40 ng/mL IFN-γ to simulate the apical periodontitis environment. Finally, the GW9662 (40 µM, MCE, Jersey City, NJ, USA) or Rosiglitazone (20 µg/mL, Macklin, Shanghai, China) was utilized to inhibit or promote the expression of PPAR γ in the in vitro models, which was application to investigate the role of PPAR γ in vitro apical periodontitis.

### 4.5. Micro-Computed Tomography (Micro-CT) Analysis

For micro-CT evaluation, the left mandibles were scanned using a micro-CT scanner (QuantumGX, Perkin Elmer, Branford, CT, USA). Images were captured with a 360° rotation, with the following settings: voltage, 90 kV; current, 88 µA; field of view, 25 mm; voxel size, 50 µm. Scanned data were reconstructed and analyzed using Mimics 17.0 software (Materialize, Leuven, Belgium). The lesion size was determined by subtracting the mean normal periodontal space in baseline controls from the total periapical radiolucent region and expressed as cubic millimeters.

### 4.6. Hematoxylin and Eosin (HE) Staining

The clinical samples and the left mandibles of each animal were fixed in 4% formaldehyde overnight. Then, the samples underwent decalcification in 10% ethylenediaminetetraacetic acid (EDTA, Boster, Wuhan, China) for a duration of 60 days. Following dehydration with ascending concentrations of ethanol (from 60% to 100%), the samples were embedded in paraffin and subsequently sectioned to a thickness of 4 µm in a mesiodistal orientation using a Leica RM 2045 microtome (Leica Microsystems, Wetzlar, Germany). The sections were stained following the manufacturer’s instructions for the HE staining kit. The results of staining were further observed and captured utilizing a light microscope (Leica Microsystems, Wetzlar, Germany).

### 4.7. Immunohistochemical (IHC) or Double Immunofluorescence (IF) Staining

The clinical samples and the right mandibles of rats in each group were collected for IHC and IF staining. The pre-treatment slices were analogous to those outlined in the HE staining. In detail, IHC staining was performed using the Streptavidin–Biotin Complex (SABC) method, following the manufacturer’s protocol from ZSJQ, China. After deparaffinization and rehydration, the 4 µm sections were subjected to an antigen retrieval solution (Boster, China) at 37 °C for 30 min. Sections were incubated with 3% H_2_O_2_ for 25 min, then blocked for 1 h with 5% BSA. Diluted primary antibodies, specifically PPAR-γ (dilution 1:200, Bioss, Beijing, China), IL-1β (dilution 1:200, Bioss, China), IL-10 (dilution 1:200, Bioss, China), Mertk (dilution 1:200, Bioss, China), and Gas 6 (dilution 1:200, Bioss, China), were applied to the sections at 4 °C for 16 h. After rinsing with PBS 3 times, the tissue slices were incubated with a secondary antibody (dilution: 1:150, ZSJQ, Beijing, China) for 30 min, and incubated with SABC for 30 min at 37 °C. The immune reactions were visualized by the DAB solution kit (ZSJQ, China). The results of IHC staining were observed and captured by a light microscope (Leica Microsystems, Germany).

For the double IF staining, following a 5% BSA blocking, the slices were incubated with primary antibodies against Mertk (Santa Cruz, Dallas, TX, USA) and c Caspase 3 (Bioss, Beijing, China). The primary antibodies were diluted at a ratio of 1:100. The secondary antibodies utilized were goat against rabbit CY3 (dilution 1:150, Boster, China) or goat against mouse FITC (dilution 1:150, Boster, China). The nucleus was counterstained with DAPI. The immunology-positive cells were examined and obtained using a confocal microscope (Olympus, Tokyo, Japan).

Semi-quantitative analysis was performed using ImageJ software (version number, v1.8.0, NIH, Bethesda, MD, USA). We used the G Power software version 3.1.9.7 (https://stats.idre.ucla.edu/other/gpower/, accessed on 30 August 2025, UCLA, USA) to verify the test power, and confirmed that at this sample size, the test power was greater than 95% and *p* < 0.05. Pearson’s correlation and linear tendency tests of PPAR-γ and the progression of CAP and macrophage efferocytosis were investigated via GraphPad 9.0 (La Jolla, CA, USA).

### 4.8. The Ratio of Efferocytosis

To investigate the ratio of efferocytosis, the co-cultured models were established as in previous research [13]. In summary, the apoptosis of HL-60 cells was labeled with CFDA SE, and the THP-1 treatment with different reagents was labeled with Dil. The CFDA and Dil positive cells were examined and obtained using a confocal microscope (Olympus, Japan). The ratio of efferocytosis was the ratio of double-positive cells. Semi-quantitative analysis was performed using ImageJ software (NIH, Bethesda, MD, USA).

### 4.9. Quantitative Real-Time PCR (qRT-PCR)

Total RNA was extracted using TRIzol reagent (Thermo Fisher Scientific, Waltham, MA, USA) based on the manufacturer’s protocol. The RNA samples were dissolved in RNase-free water and measured by a NanoDrop spectrophotometer (Thermo Fisher Scientific, Waltham, MA, USA) to examine the RNA purity and concentration. Then, 1 µg total RNA was reversed transcribed into cDNA with a RevertAid First Strand cDNA synthesis kit (Thermo Fisher Scientific, USA). qRT-PCR was conducted to analyze mRNA expression utilizing SYBR Premix ExTaq II PCR kit (TAKARA, Shiga, Japan) on an ABI PRISM 7700 (Applied Biosystems, Woburn, MA, USA). All experiments were performed in triplicate. Relative gene expression was assessed using the 2^−ΔΔCt^ method, normalizing with gapdh as the internal control. The primer sets used in this study are presented in Table 2.

### 4.10. Western Blot (WB) Analysis

The cells in each group were collected, lysed with RIPA Lysis Buffer (ZHHC, China), and centrifuged at 16,200× *g* for 10 min. The total protein concentration was quantified with a BCA protein assay kit (Beyotime Biotechnology, Shanghai, China). The protein at 35 µg was subjected to SDS-PAGE gels. The gel concentration fluctuates based on the molecular size of the target protein. Then the protein was transferred onto polyvinylidenedifluoride (PVDF) membranes (Millipore, Burlington, MA, USA). Following a 1 h blocking with 5% nonfat milk or 5% BSA (for phosphorylated protein) at room temperature, the PVDF membranes were incubated with appropriate primary antibodies at 4 °C for 18 h, subsequently followed by a 1.5 h incubation at room temperature with HRP-conjugated anti-rabbit secondary antibodies (1:5000, Boster, Wuhan, China). The protein bands were inspected with an enhanced chemiluminescence kit (Millipore, MA, USA). The Western blot results were analyzed by the ImageJ v1.8.0 software. The following primary antibodies were used: PPAR-γ (dilution 1:1000) and β-actin (dilution 1:1000, Bioss, Beijing, China). Among them, β-actin was designated as the internal control for total protein.

### 4.11. Statistical Analysis

Data are displayed as mean ± standard deviation (SD). A minimum of three repetitions of each experiment was performed. Statistical analysis was conducted with GraphPad 9.0 (La Jolla, CA, USA). First, the normality of the data was analyzed. One-way analysis of variance and Tukey’s post hoc test were used for the data conforming to normality and homogeneity of variance. For data that did not conform to normality, nonparametric tests were used to assess differences between groups. A *p*-value < 0.05 was deemed statistically significant.

## 5. Conclusions

**Figure 9:**
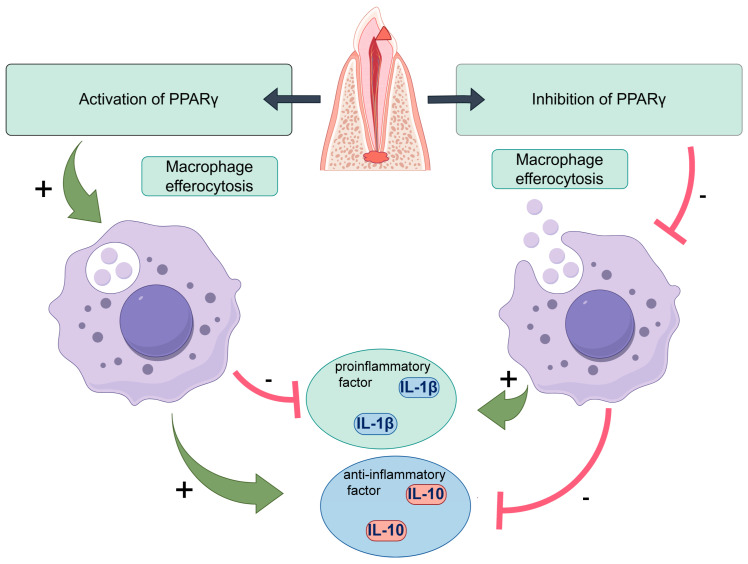
Augmentation of PPAR-γ signaling significantly attenuates inflammatory status and tissue damage in periapical lesions during CAP progression through the activation of macrophage efferocytosis. These findings offer a novel insight into a potential therapeutic approach for the treatment of CAP.

The results of this study demonstrate that the augmentation of PPAR-γ signaling significantly attenuates inflammatory status and tissue damage in periapical lesions during CAP progression through the activation of macrophage efferocytosis. These findings offer a novel insight into a potential therapeutic approach for the treatment of CAP (Figure 9).

## Figures and Tables

**Table 1:** Treatment strategy targeting PPAR-γ.

Therapeutic Strategy	Model/Study	Key Findings	Reference
Rosiglitazone (PPAR-γ agonist, synthetic drug)	Mouse model of CAP	Reduced inflammatory cytokine expression, decreased tissue damage	de Oliveira. et al. *J. Endod*. 2017 [33]
Curcumin (plant-derived polyphenol)	Mouse model of CAP	Activated PPAR-γ signaling suppressed NF-κB, mitigating periapical bone resorption	Justo MP. et al. *Int. Endod. J*. 2022 [34]

**Table 2:** The primers for qRT-PCR.

Gene	Forward Primer (from 5′ to 3′)	Reverse Primer (from 3′ to 5′)
*Gapdh*	AGAAGGCTGGGGCTCATTTG	CTTCTGACACCTACCGGGGA
*IL-1β*	CAGAAGTACCTGAGCTCGCC	GAAGCCCTTGCTGTAGTGGT
*IL-10*	GCCTTGTCTGAGATGATCCAGTT	ACAGTAGCTAAAGAAGGGACACT
*Mertk*	CCGCCCCACCTTTTCAGTAT	TTACCCCCGTCACTCCTTAC

## Data Availability

The original contributions presented in this study are included in the article. Further inquiries can be directed to the corresponding authors.

## Supplementary Materials

The following supporting information can be downloaded at: https://www.mdpi.com/article/10.3390/ijms262010157/s1.

## Abbreviations

The following abbreviations are used in this manuscript:

CAP	Chronic apical periodontitis
RSG	Rosiglitazone
