# Supplementary material for: PPAR-γ Inhibits Chronic Apical Periodontitis by Facilitating Macrophage Efferocytosis
Source: Int J Mol Sci. 2025 Oct 19;26(20):10157. doi: 10.3390/ijms262010157 (PMC12563434; doi:10.3390/ijms262010157)
Supplement: Supplementary file 1 [file ijms-26-10157-s001.zip › ijms-3910404-supplementary.pdf]

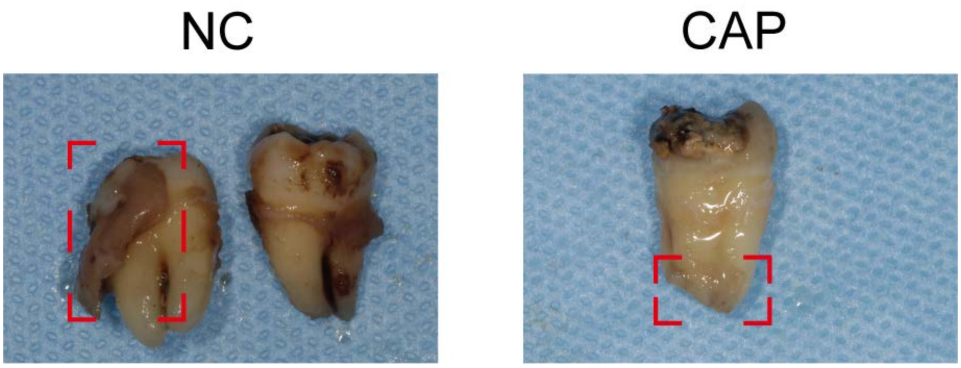

Supplementary Figure 1. The representative images of clinical samples. NC, healthy gingiva tissues, CAP, apical periodontitis tissue.

Supplementary Table 1. The demographic characteristics of the clinical samples

	Cont	PGs	RCs
**Gender**	Male: female=1:1		
**Age**	18 to 60 years old		
**Source**	Xi'an Jiaotong University Stomatological Hospital		
**Tooth position**	Gingival tissues of third molars and healthy teeth willing to be extracted for orthodontic purposes	Teeth with obvious symptoms of periapical inflammation, involving multiple teeth	
**Associated systemic diseases**	No diabetes, cardiovascular disease, smoking, or mental illness, and had not used antibiotics in the past 6 months		
**Radiological examination**	No obvious periapical transmission image	There is a radiolucent focus with irregular contours and ill-defined margins, absent a sclerotic border. This appearance is indicative of inflammatory tissue lacking a well-defined capsule.	X-ray films showed round-like or ovoid radiolucence with clear margins and a dense band of bone sclerosis (white line) around, suggesting calcification of the cyst wall formed by chronic inflammatory reaction.

Supplementary Table 2. The prognostic and demographic characteristics of the animals

	**0d**	**7d**	**14d**	**21d**	**28d**
**Gender**	**male**				
**Age**	**6 to 8 weeks old**				
**Source**	**The Laboratory Animal Center, Xi 'an Jiaotong University**				
**Environment**	**12h light/ dark cycle with stable temperature (25±2°C) and humidity (50±5%)**				
**Establishment of CAP model**	The pulp cavity of bilateral mandibular first molars were exposed via 1/4# round bur				
**Prognostic time point**	0d	7d	14d	21d	28d
**Radiological images**	No transmission images were observed in the periapical tissues.	Transmission images began to appear in the periapical region.	Periapical radiolucency began to increase	The transmission images reached a peak	The area of bone destruction tended to be stable
**Histologic Features**	No inflammatory cell infiltration and bone resorption in the periapical area	Inflammatory cell infiltration and mild bone resorption were observed in the periapical area.	All the pulp was necrotic and the apical inflammation was aggravated.	Periapical destruction reaches a peak and apical abscess may appear	In the chronic phase, the inflammatory infiltration was reduced.
